# Validation of the Primerdesign Quantitative Allele Specific Amplification Kit for the Detection of JAK2V617F Mutation

**DOI:** 10.1002/jcla.70180

**Published:** 2026-02-19

**Authors:** Deborah Vaz, Tshiphiri Netshidzivhani, Johan Potgieter

**Affiliations:** ^1^ National Health Laboratory Service ‐ Tshwane Academic Division Pretoria South Africa; ^2^ Department of Haematology, Faculty of Health Sciences University of Pretoria Pretoria South Africa

**Keywords:** JAK2V617F, polymerase chain reaction, quantitative allele specific amplification

## Abstract

**Introduction:**

Janus Kinase 2 (JAK2) is a tyrosine kinase involved in cellular signalling. A point mutation in codon 617 of JAK2 (JAK2V617F) causes a gain‐of‐function effect that stimulates proliferation of myeloid cells. The aim of this study was to evaluate the Primerdesign quantitative allele‐specific amplification (Quasa) kit for the detection of JAK2V617F.

**Methods:**

Specimens previously evaluated using real‐time PCR with melting curve analysis were analysed using the Quasa kit on the Bio‐Rad CFX 96 platform. Results of the two PCR tests were compared to evaluate the accuracy of the kit. For intra‐assay precision, samples were analysed in duplicate on the same run, and for inter‐assay variation, the same known samples were tested on three non‐consecutive days. The analytical sensitivity and quantitative analysis of the assay were determined using the World Health Organization (WHO) 1st International Reference Panel for Genomic JAK2V617F (United Kingdom Official Medicines Control Laboratory) and the United Kingdom National External Quality Assessment Service (UK NEQAS) results of their JAK2 p.val617phe (V617F) Mutation Status Programme.

**Results:**

Concordance of the qualitative test results between the test methods was 100%. Precision experiments confirmed repeatability and reproducibility. Analytical sensitivity was confirmed at 0.1%.

**Conclusion:**

The PrimerDesign Quasa kit for the detection of JAK2V617F is a sensitive and reliable assay that yields reproducible qualitative results. The kit is commercially available and has, in addition, the potential for quantitative analysis when samples are analysed in duplicate. This kit assay validation may be valuable to laboratories offering or interested in offering JAK2V617F mutational testing.

## Graphical abstract

The PrimerDesign Quasa kit demonstrates efficacy as a reliable assay for the detection of JAK2V617F mutation, exhibiting potential for quantitative analysis when samples are analysed in duplicate. This assay validation information may prove valuable to laboratories seeking to implement a simplified JAK2V617F mutational test.

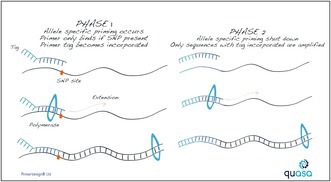

## 1 Introduction

The intracellular tyrosine kinase domain known as Janus Kinase 2 (JAK2) are necessary in performing the function of trans‐phosphorylation and in doing so, delivering a signal which activates transcription of necessary proteins [1]. Mutations within the JAK2 gene cause constitutively active signalling which is the cause for many myeloproliferative neoplasms [2]. The somatic point mutation of guanine substitution to thiamine at base 1849 in exon 14 of the JAK2 gene causes; phenylalanine to be substituted for valine in codon 617 of the JAK2 post‐transcription product (JAK2V617F) [3]. The myeloproliferative neoplasms (MPN) in which this pathogenic variant is predominantly found include: polycythaemia vera (PV) (97%), essential thrombocythaemia (ET) (57%) and primary myelofibrosis (PMF) (50%) [2]. A small percentage of atypical or unclassifiable MPN were also found to be JAK2 positive (~20%) including Hypereosinophilic syndrome (HES) (~2%). Since its discovery in 2005, the JAK2V617F mutation (JAK2‐M) has become a major diagnostic criteria for the diagnosis of Philadelphia negative myeloproliferative neoplasms [2, 3, 4]. Its presence confirms the diagnosis of PV, ET or PMF, when the other WHO diagnostic criteria are met, and therefore has a high positive predictive value. Not all patients necessitate JAK2‐M testing; a careful evaluation of blood results and additional patient history is essential to exclude secondary causes for a reactive increase in cell counts. Various predictive models are available to assess the necessity for JAK2 investigation. Among these, the JAK2‐tree prediction model is noted for its high sensitivity, albeit with limited specificity [5]. Another proposed model, the JAKPOT, demonstrates good sensitivity and improved specificity, but it is primarily applicable in cases of erythrocytosis and may not include patients with suspected essential thrombocythemia (ET) or primary myelofibrosis (PMF) who present with isolated elevations in platelet or white cell counts [6].

The presence of JAK2‐M can aid in the differentiation between MDS/MPN with neutrophilia and PMF, when diagnosis can sometimes be a difficult one to make [7]. Identification of this mutation along with many others has improved the diagnostic classification of these disorders in the World Health Organization (WHO) 2022 classification and the International Consensus Classification (ICC) [7]. Mutation detection confirms clonality and may have prognostic value with the possibility of offering specific targeted therapy such as JAK2 specific kinase inhibitors, for example, Ruxolitinib [8, 9]. The identification of the JAK‐M in a patient with ET, has been shown to predispose these patients to a prothrombotic phenotype of the disease and therefore encouraging the early use of aspirin or P2Y12 inhibitors [10]. Detection of the JAK2V617 mutational load can also be used for monitoring disease progression [2].

Various methodologies for testing this mutation have been evaluated and compared in terms of accuracy and sensitivity. The allele‐specific PCR (AS‐PCR) techniques have demonstrated superior performance, with detection limits reaching 0.1% of the variant allele burden, in contrast to melting curve analysis, which ranges from 1% to 10% [11, 12, 13]. When compared to Pyrosequencing or Next Generation Sequencing (NGS), AS‐PCR shows concordance with both methods, with NGS providing robust allele burden quantification. However, AS‐PCR maintains superior sensitivity detection compared to pyrosequencing or NGS, which have detection limits of 20% and 2%, respectively [14, 15]. In the context of 3D digital PCR, it has been found to be more reproducible in quantifying the JAK2V617F mutational load compared to real‐time quantitative PCR (RQ‐PCR) [16]. This reproducibility is attributed to its ability to measure allele burden even at low mutation concentrations and its independence from standard curves. Recent studies comparing NGS and Digital PCR have shown a strong correlation and excellent allele burden quantification between the two, albeit with higher levels of detection needed [17].

Quantitative allele specific amplification (Quasa) is a super selective mastermix that can increase the specificity of allele specific polymerase chain reaction (PCR) up to 100‐fold by improved stringency during allele specific priming [18]. The Quasa primers are modified in that they have a sequence independent ‘tag’ at the 5′ end. The JAK2V617F Quasa commercial kit by Primerdesign employs a modified master mix, hydrolysis probe, and two modified primers: a V617F single nucleotide polymorphism (SNP) detection primer and a wild‐type (WT) detection primer. PrimerDesign claims an analytical sensitivity of 0.1% for the JAK2V617F Quasa kit. In addition, the kit allows for quantification in that the proportion of mutant amplicons is expressed as a percentage relative to the WT amplicons. The validation of this kit is shown here, with accuracy and reliability shown and analytical sensitivity as per the manufacturer's claims.

## 2 Materials and Methods

### 2.1 Samples and DNA Extraction

This study was approved by the NHLS via the Academic Affairs, Research, and Quality Assurance (AARQA) department to access data on the laboratory information system. Peripheral blood was collected in evacuated ethylenediaminetetraacetic acid (EDTA) collection tubes (*n* = 34) as part of the workup of patients with suspected MPN at the Haematology Clinic of the Steve Biko Academic Hospital (SBAH). Genomic DNA was extracted from 200 μL of peripheral blood using a High Pure PCR template preparation kit (Roche, Switzerland) according to the manufacturer's instructions. The concentration and purity of genomic DNA were analysed using a Genova Nano micro‐volume spectrophotometer (Jenway, Staffordshire, UK). Extracted DNA was aliquoted and stored at −20°C until analysis.

### 2.2 Real‐Time PCR Assay

**FIGURE 1:**
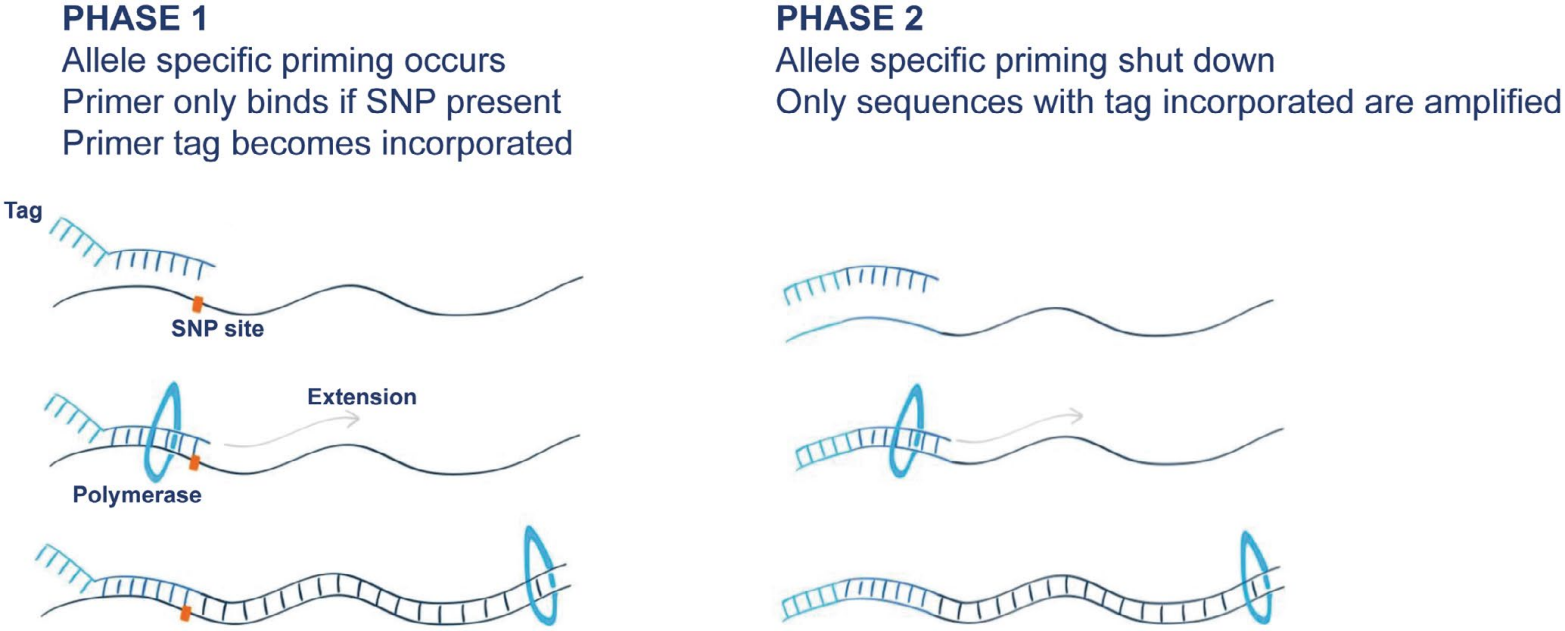
Schematic representation of the method of action of the modified primers used by the Quasa assay kit.

A quantitative allele‐specific amplification (Quasa) real‐time PCR kit (Primerdesign, UK) was used to amplify DNA. Primers were designed such that the 3′ terminal base overlies the mutation site; thus, wild‐type (WT) primers confer 100% specificity to the wild‐type sequence but have a single base mismatch with the mutated sequence. The tag was incorporated into the amplicon during the first round of PCR and was then present in the amplicon for subsequent cycles as shown in phase one of Figure 1. This means that the tagged primers will prime preferentially on this template and drive the amplification of the correct sequence forward, as shown in phase two of Figure 1.

The PCR assay mixture, which had a single nucleotide polymorphism (SNP) V617F primer/probe and the wild‐type (WT) primer/probe, was added to each reaction, followed by the addition of 5 μL of 5 ng/μL of DNA sample [Table S1, Appendix S1]. Real‐time PCR assays were performed on a CFX 96 thermal cycler (Bio‐Rad, Germany) under the following conditions: enzyme activation for 2 min at 95°C followed by two stages of amplification. The first stage included five cycles of denaturation at 95°C for 10 s, annealing at 50°C for 15 s, and extension at 72°C for 15 s. The second stage of amplification consisted of 40 cycles of denaturation at 95°C for 10 s, annealing at 60°C for 30 s, and extension at 72°C for 15 s [Table S2, Appendix S1].

### 2.3 Analysis Calculation Method

**FIGURE 2:**
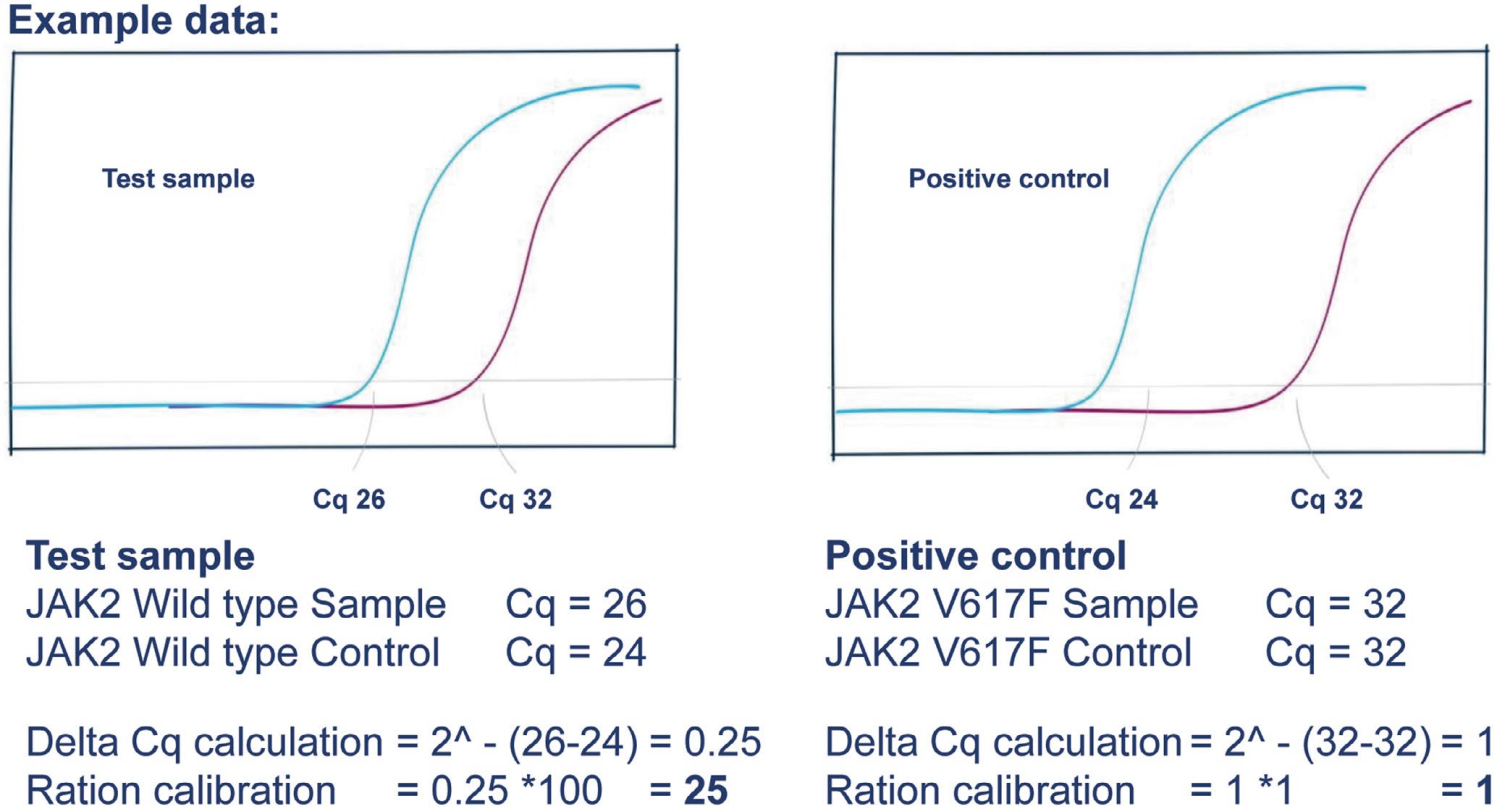
Step 1 PrimerDesign Quasa kit calculation of percentages for samples and control data.

**FIGURE 3:**
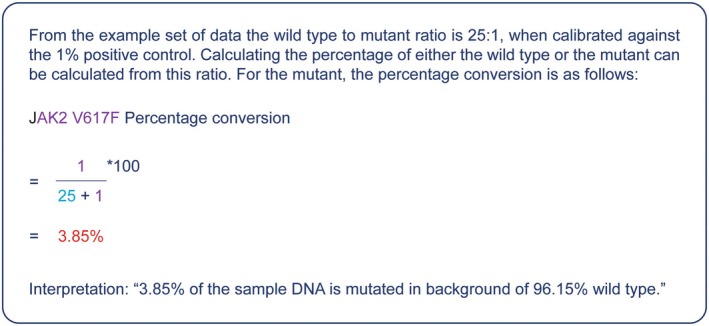
Step 2 PrimerDesign Quasa kit calculation of percentages for mutant and wild type DNA.

The percentage of SNP present in the sample was calculated using the delta Cq method, with anything above 0.1% considered positive. The proportions of SNP and WT in the sample were corrected by reference to a positive control standard, where the SNP was present at a known proportion of 1%. The calculations are performed in two stages. First, the delta Cq values were used to calculate the relative detection levels between the biological sample and the 1% control for both the wild type and mutant, as shown in Figure 2. The relative amounts were converted to percentages as shown in Figure 3.

### 2.4 Analysis of Accuracy

The Patient samples were sent to the reference referral laboratory of the Department of Haematology and Molecular Medicine at the Charlotte Maxeke Johannesburg Academic Hospital (CMJAH) for routine JAK2‐M screening. The referral laboratory performed a real‐time PCR assay with fluorescent resonance energy transfer (FRET) hybridisation probes and melt curve analysis on a LightCyclerTM 2.0 (Roche). The extracted DNA was then used in parallel with the Quasa kit on the Bio‐Rad CFX96 real‐time PCR instrument, and the results obtained were compared to those obtained by the reference laboratory. Qualitative accuracy was further confirmed by the assessment of performance in EQA, inclusive of the analysis of 20 survey samples. To evaluate the quantitative analysis, the World Health Organization (WHO) standards and the United Kingdom National External Quality Assessment Service (UK NEQAS) results of their JAK2 p.val617phe (V617F) mutation status program were used for the comparative analysis. All samples of the UK NEQAS and WHO standard panel specimens were analysed in duplicate using both the V617F probe and the wild‐type probe of the PrimerDesign assay, and the percentage of mutant DNA was calculated.

### 2.5 Analysis of Precision

Intra‐ and inter‐assay precision were evaluated by performing a real‐time PCR assay on samples that were known to be positive and negative for the JAK2‐M. For qualitative intra‐assay precision, nine samples were analysed in duplicate in the same run, and for inter‐assay variation, eight samples were evaluated on three non‐consecutive days. The analytical sensitivity of the assay was determined using the WHO 1st International Reference Panel for Genomic JAK2V617F (United Kingdom Official Medicines Control Laboratory). This international standard includes a panel of seven freeze‐dried genomic DNA samples that were assigned values for JAK2V167F as a percentage of total JAK2.

### 2.6 Analytical Sensitivity and Specificity

Analytical sensitivity was assessed by analysing mutant DNA diluted to less than 0.1%. The WHO standard of 1% was diluted with wild‐type DNA to 0.1%, and the WHO sample 15/170 (0.03%) was used to represent a mutant concentration below 0.1%. The accepted criterion was the ability of the assay to detect the mutation at a level of 0.1% (as claimed by the manufacturer) but not at a level of 0.03%. Analytical specificity was assessed by analysing specimens that were positive or negative for the mutation. The accepted percentage of analytical specificity was 100%.

### 2.7 Clinical Comparison

The samples tested for JAK2‐M were analysed alongside the patients' age, biological sex and complete blood counts, with both positive and negative outcomes being reviewed. A total of 207 samples were evaluated against the JAK2‐tree prediction criteria to assess the model's efficacy in determining the necessity for JAK2 testing. The model utilises the full blood counts (FBC) to determine whether the patient should have a JAK2 test performed. This includes a haemoglobin level of 16 g/dL for female patients and 16.5 g/dL for male patients, or a platelet count of > 350 × 10^9^/L, or a white cell count of > 7 × 10^9^/L. Patients correctly identified by the JAK2‐tree model for JAK2‐M testing (true positives) and those incorrectly identified (false positives) were recorded. Similarly, patients correctly not indicated for testing (true negatives) and those incorrectly not indicated (false negatives) were also documented. This data was subsequently utilised to calculate the sensitivity, specificity, and positive and negative predictive values of the JAK2‐tree prediction model.

### 2.8 Data and Statistical Analysis

Descriptive statistics with means and standard deviations were calculated for continuous variables and frequencies and proportions for categorical variables. The correlation between the precision tests was analysed using Pearson's correlation; the closer the value was to 1 or −1, the stronger the relationship between the two values. The concordance correlation coefficient was calculated as a measure of the correlation between WHO and PrimerDesign values. Linear regression was used to graphically illustrate the WHO sample values in comparison to the PrimerDesign values. The intercept and slope are reported with 95% confidence intervals (CIs). Statistical analyses were conducted using STATA 15. Statistical significance was set at 5%.

The Bland–Altman difference plot was used to graphically illustrate the WHO sample values in comparison to the PrimerDesign values. Participation in the UK NEQAS JAK2 p.val617phe (V617F) mutation status program allowed for evaluation of the JAK2 mutant allele burden. The robust mean, standard deviation (SD), and reference range for each submission are included.

## 3 Results

### 3.1 Analysis of Accuracy

**FIGURE 4:**
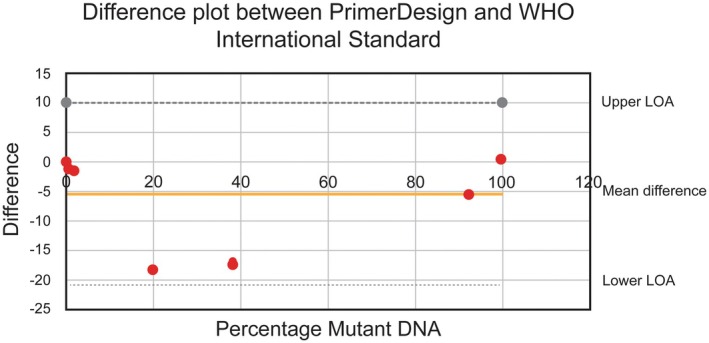
Bland Altman difference plot showing agreement between percentage of mutant DNA measured by PrimerDesign and assigned percentage of the respective WHO standards (Upper limit of agreement (LOA) = 9.9; lower LOA = −20.8). The difference plot demonstrated a positive bias, with all PrimerDesign percentages exceeding those of the WHO [Table S4, Appendix S1]. Nevertheless, all values remained within the accepted limits of agreement (LOA).

Qualitative: Of the 34 specimens analysed using the Quasa kit, 15 were positive and 19 were negative. The qualitative results were compared with those obtained in the reference laboratory and showed a 100% concordance [Table S3, Appendix S1]. Quantitative: When the percentage of mutant DNA (Quasa, PrimerDesign) was calculated and compared to the assigned percentage of the respective WHO standards, the following was found: For the WHO sample code 15/164 (100%), the PrimerDesign assay estimated mutant DNA at 99% V617F. For codes 15/246 (89.5%), 15/244 (29.6%), 15/166 (10.8%), and 15/168 (1.0%), the PrimerDesign assay overestimated the percentage of mutant DNA [Table S4, Appendix S1]. The PrimerDesign assay did not detect mutant DNA in WHO sample codes 15/170 (0.03%) or 15/172 (0%). The agreement between the values is shown in the difference plot as seen in Figure 4.

Although a positive bias was observed for most of the WHO sample results, all values were plotted within the accepted limits of agreement. Linearity between the PrimerDesign and assigned WHO standards is shown (*r* ^2^ = 0.972 [95% confidence interval: 0.79–1.15]; *Y* intercept: −4.5). Linear Regression between WHO percentages and the PrimerDesign mean values is shown in Figure S1 of the Appendix S1. Quantification of the percentage JAK2 mutation load in all survey specimens received from the UK NEQAS JAK2 p.val617phe (V617F) mutation status program was within consensus. The allele burden percentage of the JAK2, measured using the Quasa kit, is shown to be within acceptable ranges of the UK NEQAS, summary of the performance of the assay in the UK NEQAS JAK2V617F mutation status program is shown in Table 1. To date, all qualitative and quantitative results have been in consensus [Table S5, Appendix S1].

**TABLE 1:** Summary of the performance of the Primerdesign Quasa assay in the UK NEQAS JAK2V617F mutation status program. Interpreted with the robust mean and SD value. The range was calculated.

UK NEQAS Sample number	JAK2 mutant detected^†^ (%)	Robust mean	Robust SD^‡^	*p*	CI (95%)
184	47	47.6	9.58	0.9501	28.4–66.8
176	5	7.38	2.35	0.3112	2.74–12.1
186	3	7.16	2.43	0.0869	2.3–12.0
177	43	52.8	6.82	0.1507	39.2–66.4
172	35.5	38.4	7.99	0.7166	22.7–54.0
179	2.7	5.94	1.85	0.0799	2.24–9.6
Eduk	0.3	0.94	0.43	0.1367	0.08–1.8

^†^ Mean value of specimen, which was run in duplicate.

^‡^ Standard deviation.

### 3.2 Analysis of Precision

Both the qualitative results and the percentage estimation of allele burden were repeatable for all samples [Table S6, Appendix S1]. Apart from the variability of the duplicate PrimerDesign assay estimation for code 15/166 (10.8%), repeatability was observed for the quantification of all other codes. Pearson's correlation coefficient between duplicate results was 0.9 (with an absolute mean difference of 0.4). Most samples yielded reproducible results on different days, as indicated by a Pearson's correlation of 0.9. However, the absolute mean difference was 2.6, mostly because of the moderate variation in percentages estimated between the days. The difference between these values was not statistically significant (*p* = 0.37). The inter‐assay precision of the Quasa Kit yielded acceptable qualitative results. The quantitative results were comparable [Table S7, Appendix S1].

### 3.3 Analytical Sensitivity and Specificity

The analytical sensitivity of the PrimerDesign assay was 0.1%. The World Health Organization standard sample 15/168 (1.0%) was diluted to 0.1% with sample 15/172 (0%) and the mixture was analysed repeatedly using the PrimerDesign assay. The results were consistently positive. The WHO standard sample 15/170 (0.03%) was also analysed, and the results were consistently negative. Analytical specificity was 100%. All specimens known to be negative for the mutation, as well as the WHO sample 15/172 (0%), tested negative for JAK2‐M.

### 3.4 Clinical Comparison

A total of 207 samples tested for JAK2‐M were reviewed with 156 negative and 51 positive. Results of JAK2‐M testing with patient demographics is shown in Table 2. The majority of the positive results were seen in male patients in the older age group cohort (65–90). Patients who fulfilled the JAK2‐tree criteria for JAK2‐M testing were classified as indicated, whereas those who did not meet the criteria were classified as not indicated. A total of 47 patients were truly indicated, meeting the required elevated levels of haemoglobin, platelet count, or white cell count, while 26 patients were truly not indicated, as they did not meet the necessary FBC levels. The sensitivity of this predictive model was 92% (95% CI: 84.78% to 99.54%), with a negative predictive value of 87% (95% CI: 74.50% to 98.83%) [Table S8, Appendix S1].

**TABLE 2:** Results of JAK2‐M testing with patient demographics, *n* = number of patients.

	Female (*n*)			Female total (*n*)	Male (*n*)			Male total (*n*)
Age groups	15–39	40–64	65–90		15–39	40–64	65–90	
Negative	17	28	10	55	28	49	24	101
Positive	3	7	8	18	2	14	17	33
Grand total				73				134

## 4 Discussion

JAK2 plays a critical role in the signal transduction of various cytokines during haematopoiesis. Mutations in JAK2 that result in unregulated signalling can lead to a range of myeloproliferative neoplasms, making it a crucial target for testing [2, 7]. The objective of this study was to validate the PrimerDesign JAK2V617F Quasa kit. This assay was selected based on the manufacturer's assertions regarding its performance and user‐friendliness. Our findings demonstrated 100% concordance in accuracy [Table S3, Appendix S1], with commendable qualitative precision and an analytical sensitivity of 0.1%. Previous investigations comparing the analytical sensitivity of JAK2V617F using DNA sequencing and PCR‐based techniques have indicated that AS‐PCR assays exhibit sensitivity (0.5%–1%) superior to both melting curve analysis (1%–10%) and sequencing (5%–10%) [11, 13, 14]. The Quasa modified primers ensured 100% specificity in several ways. First, each of the wild‐type and mutant primers binds specifically to the sequence at hand with a single base mismatch, which prevents incorrect binding. Therefore, the wild‐type primer will not bind to a mutant DNA sequence because of a single‐base mismatch and vice versa. Second, the lower melting temperatures of the primers make the incorrect binding as unstable as possible, resulting in an inability to amplify. Therefore, detection of the amplicon at this site will not occur. Lastly, there is a sequence independent ‘tag’ which is attached to the primer at the 5′ end, which will be incorporated into the amplicon after the first round of PCR. This allowed the tagged primers to proceed with amplification and preferentially bind to the same primer/tag sequence.

In evaluating mutation allele burden, both 3D digital PCR and NGS have exhibited superior performance compared to AS‐PCR [15, 16, 17]. The results of our quantitative assay indicate this, such that the values did not align with the majority of the WHO sample results. They displayed a positive bias throughout; however, all values remained within the accepted limits of agreement [Figure 4]. Monitoring residual disease in JAK2‐positive MPN has become integral to standard management following the approval of Ruxolitinib in November 2012. This represents the first JAK2 inhibitor available on the market, developed merely 6 years after the mutation's discovery [19]. A higher allele burden of the JAK2V617F mutation in ET or PV is correlated with an increased risk of thrombosis and cardiovascular events [20]. An allele burden exceeding 50% in PV patients is associated with an elevated risk of progression to myelofibrosis [21]. The JAK2 inhibitor is not widely available in our setting, and consequently, disease burden monitoring is not yet conducted.

An investigation for the JAK2‐M may not be recommended for all patients unless they meet specific FBC criteria. We undertook a comparison of specimens tested for JAK2‐M with their corresponding FBC results to evaluate whether they satisfy the criteria established by the JAK2‐tree prediction model [5]. We selected this prediction model over the JAKPOT model because it is specifically designed to identify patients requiring JAK2 investigation in the context of erythrocytosis [6]. Our findings were consistent with those of the JAK2‐tree prediction model, demonstrating high sensitivity [92% (95% CI: 84.78% to 99.54%)] and a high negative predictive value [87% (95% CI: 74.50% to 98.83%)]. This is attributable to the model's capacity to confidently exclude patients who do not meet any FBC criteria from requiring a JAK2 investigation. As anticipated and noted by the authors of the JAK2‐tree model paper, the specificity and positive predictive values were low.

A new diagnostic assay needs to be validated for use as part of a functioning quality management system. This is also a requirement for the standard employed to ensure the delivery of accurate and reliable results (International Organization for Standardization [ISO] 15189). The Clinical and Laboratory Standards Institute (CLSI) provides further guidance by means of the Quality Management System: A Model for Laboratory Services (GP26‐A4) document [22]. The goal of method validation in a molecular diagnostic assay is to ensure the investigation is fit for purpose and can be performed with confidence by the laboratory [23]. To reach this goal successfully, each necessary step needs to be followed, evaluated and documented. As part of the introduction of the new test, a standard operating procedure (SOP) must be drafted for laboratory technologists to guide the necessary steps to follow when using the diagnostic kit. Ongoing quality control measures need to be maintained, including internal quality controls and external quality proficiency tests. Testing of new reagents and routine maintenance of instruments are also required. Laboratory staff continued competency assessments should be conducted regularly [22].

The Quasa assay is thus comparable to other validated methodologies; however, the quantitative analysis results, while closely related, were not as precise, indicating that the quantitative accuracy of the kit may require further validation studies. In the published WHO International Reference Panel for Genomic JAK2V617F, variations were observed among the methods employed by different institutions, yet a consensus percentage value was established for each reference panel [24]. The discrepancies in values were attributed to the diverse methods, reagents, and operators across facilities, complicating the standardization of quantitative molecular markers. Based on the findings of this study, the Quasa kit is utilized at the NHLS (TAD) Haematology laboratory for the qualitative detection of the JAK‐M, but not for assessing mutational allele burden.

## 5 Conclusion

The PrimerDesign Quasa kit exhibited complete concordance with melting curve analysis in the detection of JAK2‐M, while also demonstrating superior analytical sensitivity at 0.1%. The test yielded results that were both consistent and reproducible. This method is recommended for patients who meet the FBC criteria as outlined by the JAK2‐tree prediction model for the routine detection of JAK2‐M at diagnosis. The implementation of this investigative method has enhanced service delivery to clinicians and patients in our setting.

## Author Contributions

Dr. Deborah Vaz contributed to the design and implementation of the research and the writing of the manuscript. Ms. Tshiphiri Netshidzivhani contributed to the analysis of the results and to editing of the manuscript. Prof. Johan Potgieter conceived the original, contributed to the writing of the manuscript, and supervised the project.

## Disclosure

The work described in this manuscript was presented in part as a poster at the International Society of Lab Haematology (ISLH) Congress in 2022. The illustrations provided are sourced from the Quasa handbook, and PrimerDesign has granted permission for their use. The images were adjusted by Ms. M. Booyens from Creative Studios at the University of Pretoria.

## Ethics Statement

Ethics approval was granted by the Faculty of Health Sciences Research Ethics Committee of the University of Pretoria (Protocol number 33/2019).

## Conflicts of Interest

The authors declare no conflicts of interest.

## Supporting information


**Appendix S1:** jcla70180‐sup‐0001‐SupplementaryAppendix.docx.

## Data Availability

The data that supports the findings of this study are available in the Supporting Information of this article. Additional data may be obtained from the corresponding author upon reasonable request. The Appendix S1 provides methodological and analytical data supporting this study's findings. This includes PrimerDesign Quasa kit PCR protocols, comparative accuracy, precision, and analytical sensitivity results, WHO reference standard analyses, UK NEQAS performance data, and additional statistical analyses, including linear regression of WHO‐assigned versus measured values.
