# Supplementary material for: Validation of the Primerdesign Quantitative Allele Specific Amplification Kit for the Detection of JAK2V617F Mutation
Source: J Clin Lab Anal. 2026 Feb 19;40(6):e70180. doi: 10.1002/jcla.70180 (PMC13042390; doi:10.1002/jcla.70180)
Supplement: Supplementary file 1 — Appendix S1: jcla70180‐sup‐0001‐SupplementaryAppendix.docx. [file JCLA-40-e70180-s001.docx]

Supplementary Appendix

**Methods**

Table 1: PrimerDesign Quasa® kit PCR preparation

| Reagent | Volume per reaction (µL) |
| --- | --- |
| 2x Precision Quasa Mastermix | 10 |
| Primer/probe mix | 1 |
| RNAse/DNAse free water | 4 |
| Total volume | 15 |
| Added DNA template | 5 |
| Final Volume | 20 |

Table 2: PrimerDesign Quasa® kit amplification protocol

|  | Step |  | Time (sec) | Temp (ºC) |
| --- | --- | --- | --- | --- |
|  | qPCR Enzyme Activation |  | 120 | 95 |
| 5 Cycles | Denaturation |  | 10 | 95 |
|  | Annealing |  | 15 | 50 |
|  | Extension |  | 15 | 72 |
| 40 Cycles | Denaturation |  | 10 | 95 |
|  | Annealing* |  | 30 | 60 |
|  | Extension |  | 15 | 72 |
| * Detection of fluorogenic data through the FAM channel. | |  | |  |

**Results**

Table 3: Accuracy of the PrimerDesign assay for detection of the JAK2V617F mutation compared to that of the reference method (Melting Curve Analysis)

| # | PrimerDesign Quasa® assay | Reference method |
| --- | --- | --- |
| A01 | Positive | Positive |
| A02 | Negative | Negative |
| A03 | Positive | Positive |
| A04 | Negative | Negative |
| A05 | Positive | Positive |
| A06 | Negative | Negative |
| A07 | Negative | Negative |
| A08 | Positive | Positive |
| A09 | Negative | Negative |
| A10 | Positive | Positive |
| D01 | Positive | Positive |
| D02 | Negative | Negative |
| D03 | Negative | Negative |
| D04 | Negative | Negative |
| D05 | Positive | Positive |
| D06 | Negative | Negative |
| D07 | Positive | Positive |
| D08 | Positive | Positive |
| D09 | Positive | Positive |
| D10 | Positive | Positive |
| D11 | Positive | Positive |
| D12 | Positive | Positive |
| E01 | Positive | Positive |
| E02 | Positive | Positive |
| E03 | Negative | Negative |
| E04 | Negative | Negative |
| E05 | Negative | Negative |
| E06 | Negative | Negative |
| E07 | Negative | Negative |
| E08 | Negative | Negative |
| E09 | Negative | Negative |
| E10 | Negative | Negative |
| E11 | Negative | Negative |
| E12 | Negative | Negative |

Table 4: WHO comparative results. The PrimerDesign assay was run in duplicate, and a mean value was established.

|  | 15/172 | 15/170 | 15/168 | 15/166 | 15/244 | 15/246 | 15/164 |
| --- | --- | --- | --- | --- | --- | --- | --- |
| WHO JAK2V617F % | 0 | 0.03 | 1.0 | 10.8 | 29.6 | 89.5 | 100 |
| PrimerDesign | 0 | 0 | 2.5 | 29 | 47 | 95 | 99.8 |

*Figure 1:* *Linear Regression between WHO percentages and the PrimerDesign mean values. All values were plotted within the accepted limits of agreement.*


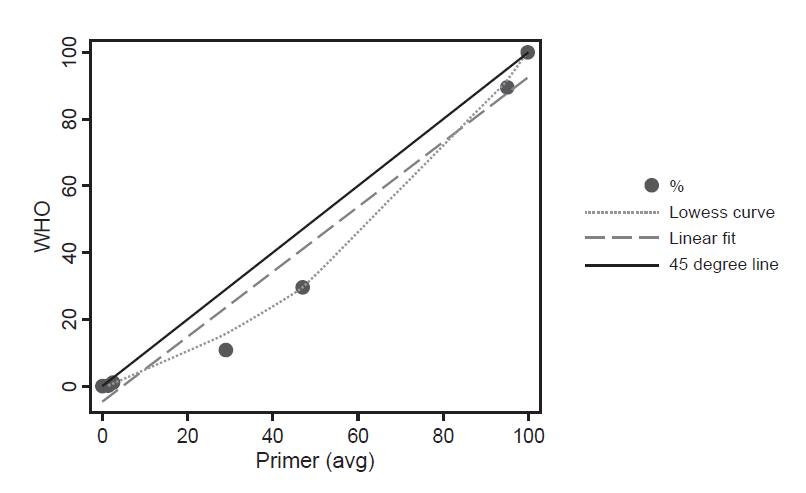


Table 5: Summary of the performance of the Primerdesign Quasa assay in the UK NEQAS JAK2V617F mutation status program – qualitative results.

|  | TAD Haematology result | Consensus result |
| --- | --- | --- |
| Sample # |  |  |
| 159 | mutation detected | mutation detected |
| 160 | mutation detected | mutation detected |
| 161 | no mutation detected | no mutation detected |
| 162 | no mutation detected | no mutation detected |
| 163 | mutation detected | mutation detected |
| 164 | mutation detected | mutation detected |
| 165 | no mutation detected | no mutation detected |
| 166 | mutation detected | mutation detected |
| 167 | mutation detected | mutation detected |
| 168 | no mutation detected | no mutation detected |
| 169 | mutation detected | mutation detected |
| 170 | mutation detected | mutation detected |
| Edu1 | mutation detected | mutation detected |
| 172 | mutation detected | mutation detected |
| 176 | mutation detected | mutation detected |
| 177 | mutation detected | mutation detected |
| 179 | mutation detected | mutation detected |
| 184 | mutation detected | mutation detected |
| 186 | mutation detected | mutation detected |
| Eduk | mutation detected | mutation detected |

Table 6: Intra-assay precision results

|  | TEST 1 | | TEST 2 | |
| --- | --- | --- | --- | --- |
| Specimen # | Quantitative (%) | Qualitative | Quantitative (%) | Qualitative |
| A01 | 93 | Positive | 93 | Positive |
| A02 | 0 | Negative | 0 | Negative |
| A03 | 5 | Positive | 5 | Positive |
| A05 | 44 | Positive | 47 | Positive |
| A06 | 0 | Negative | 0 | Negative |
| A08 | 88 | Positive | 87 | Positive |
| D03 | 0 | Negative | 0 | Negative |
| D04 | 0 | Negative | 0 | Negative |
| D06 | 0 | Negative | 0 | Negative |

Table 7: Inter-assay precision results

| Specimen # | A01 | A03 | A05 | A08 | D03 | D04 | D06 | D08 |
| --- | --- | --- | --- | --- | --- | --- | --- | --- |
| Day 1  Quantitative (%) | 93 | 5 | 47 | 87 | 0 | 0 | 0 | 79 |
| Day 1 Qualitative | Positive | Positive | Positive | Positive | Negative | Negative | Negative | Positive |
| Day 2  Quantitative (%) | 98 | 2 | 50 | 96 | 0 | 0 | 0 | 80 |
| Day 2 Qualitative | Positive | Positive | Positive | Positive | Negative | Negative | Negative | Positive |
| Day 3  Quantitative (%) | 96 | 3 | 45 | 92 | 0 | 0 | 0 | 76 |
| Day 3 Qualitative | Positive | Positive | Positive | Positive | Negative | Negative | Negative | Positive |

Table 8: Results of the JAK2-M tested samples based on inclusion criteria defined by the JAK2-Tree Predictive Model.

|  | True positive (n) | False positive (n) | Total (n) |
| --- | --- | --- | --- |
| JAK2-tree indicated testing | 47 | 130 | 177 |
|  | True negative (n) | False negative (n) | Total (n) |
| JAK2-tree not indicated to test | 26 | 4 | 30 |
